# Resistance to a Nucleoside Analog Antiviral Drug from More Rapid Extension of Drug-Containing Primers

**DOI:** 10.1128/mBio.03492-20

**Published:** 2021-02-09

**Authors:** Han Chen, Jessica L. Lawler, David J. Filman, James M. Hogle, Donald M. Coen

**Affiliations:** a Department of Biological Chemistry and Molecular Pharmacology, Blavatnik Institute, Harvard Medical School, Boston, Massachusetts, USA; Columbia University/HHMI

**Keywords:** DNA replication, antiviral drugs, drug resistance mechanisms, ganciclovir, human cytomegalovirus, nucleoside analogs, polymerases

## Abstract

Nucleoside analogs are mainstays of antiviral therapy. Although resistance to these drugs hinders their use, understanding resistance can illuminate mechanisms of the drugs and their targets. Certain nucleoside analogs, such as ganciclovir (GCV), a leading therapy for human cytomegalovirus (HCMV), contain the equivalent of a 3′-hydoxyl moiety, yet their triphosphates can terminate genome synthesis (nonobligate chain termination). For ganciclovir, chain termination is delayed until incorporation of the subsequent nucleotide, after which viral polymerase idling (repeated addition and removal of incorporated nucleotides) prevents extension. Here, we investigated how an alanine-to-glycine substitution at residue 987 (A987G), in conserved motif V in the thumb subdomain of the catalytic subunit (Pol) of HCMV DNA polymerase, affects polymerase function to overcome delayed chain termination and confer ganciclovir resistance. Steady-state enzyme kinetic studies revealed no effects of this substitution on incorporation of ganciclovir-triphosphate into DNA that could explain resistance. We also found no effects of the substitution on Pol’s exonuclease activity, and the mutant enzyme still exhibited idling after incorporation of GCV and the subsequent nucleotide. However, despite extending normal DNA primers similarly to wild-type enzyme, A987G Pol more rapidly extended ganciclovir-containing DNA primers, thereby overcoming chain termination. The mutant Pol also more rapidly extended RNA primers, a previously unreported activity for HCMV Pol. Structural analysis of related Pols bound to primer-templates provides a rationale for these results. These studies uncover a new drug resistance mechanism, potentially applicable to other nonobligate chain-terminating nucleoside analogs, and shed light on polymerase functions.

## Abstract (precis)

While resistance to antiviral drugs can hinder their clinical use, understanding resistance mechanisms can illuminate how these drugs and their targets act. We studied a substitution in the human cytomegalovirus (HCMV) DNA polymerase that confers resistance to a leading anti-HCMV drug, ganciclovir.

## INTRODUCTION

Nucleoside analogs are mainstays of antiviral therapy and are important for anti-cancer chemotherapy (1). In general, these compounds are phosphorylated intracellularly into drug triphosphates that compete with natural nucleotides to inhibit polymerases and/or are incorporated into genomes where they often serve as chain terminators. Resistance to these drugs can limit their effectiveness in the clinic. Understanding resistance mechanisms can shed light on biochemical and biological mechanisms of the drugs, their targets, and the organisms encoding those targets.

The DNA polymerases of herpesviruses are prototypes for family B DNA polymerases, including human DNA polymerases α, δ, and ε, and are also targets of leading drugs for treating herpesvirus infections. The herpesvirus human cytomegalovirus (HCMV) is a common opportunistic pathogen that can cause significant morbidity and mortality, particularly in immunocompromised patients and newborns (2). First-line therapies against HCMV include the nucleoside analog ganciclovir (GCV) and its prodrug, valganciclovir (3). Additionally, ganciclovir in combination with gene delivery of an enzyme that phosphorylates it has been explored as a cancer therapy (4) and as a suicide system to eliminate harmful stem cells following their transplantation (5).

GCV is converted to its triphosphate (GCV-TP), which serves as both a competitive inhibitor and a substrate for the targeted DNA polymerase (6–8). However, unlike a number of nucleoside analogs, GCV is not an obligate chain terminator due to the equivalent of a 3′ hydroxyl group in its sugar moiety. Instead, HCMV DNA polymerase (like other family B DNA polymerases) terminates DNA synthesis after incorporating GCV and the next incorporated (N + 1) nucleotide (6, 9–11) (delayed, nonobligate chain termination).

Prolonged use of GCV can lead to selection for resistance mutations in the gene encoding the catalytic subunit (Pol) of HCMV DNA polymerase that are often associated with treatment failures (12). About half of these GCV resistance (GCV^r^) mutations affect residues in or near motifs conserved among 3′–5′ exonuclease (Exo) domains (13). At least three of these mutations impair Exo activity, thereby overcoming chain termination at the N + 1 position by eliminating idling (repeated incorporation of the N + 2 nucleotide followed by its rapid removal), resulting in continued DNA synthesis after incorporation of GCV and the N + 1 nucleotide (14). Moreover, once the N + 1 nucleotide is incorporated, it is not detectably excised, even by wild-type (WT) Pol (14). Thus, studies of Exo mutant HCMV Pols helped elucidate how GCV-TP induces delayed, nonobligate chain termination.

This unusual drug resistance mechanism for GCV^r^ Exo mutants inspired us to investigate examples of GCV^r^ mutations that affect the polymerase domain of HCMV Pol (13), which, like other family B DNA polymerases, contains finger, palm, and thumb subdomains. One GCV^r^ mutation, A987G, which confers ∼5-fold resistance to GCV, alters highly conserved region V, which lies within the thumb subdomain of HCMV Pol based on the structure of the closely related herpes simplex virus 1 (HSV-1) Pol (15). This substitution, which was found in the first reported GCV^r^ mutant (16, 17), has also frequently been detected in clinical isolates associated with treatment failures in transplant recipients (18–20). Structures of family B polymerases bound to DNA primer-templates show that residues in conserved region V directly contact the backbone of the DNA primer (21–23), but functional studies of the effects of mutations in this region have been limited (24). Thus, studying the mechanism of GCV^r^ conferred by the HCMV A987G substitution may inform our understanding of polymerases across all three domains of life.

Interestingly, whether herpesvirus Pols can utilize RNA primers, which are crucial for initiation of DNA synthesis and for lagging-strand synthesis, has been controversial (25–27). To our knowledge, no one has examined utilization of RNA primers by HCMV DNA polymerase.

Our initial hypothesis was that the A987G substitution would cause GCV^r^ by altering binding or incorporation of GCV-TP into primer-template, much as the acyclovir resistance (ACV^r^) N961K substitution in region V of HSV-1 Pol decreases both binding and incorporation of ACV-TP (28). Alternative hypotheses for resistance mechanisms included increased excision of incorporated GCV, similar to substitutions in the bacteriophage Φ29 DNA polymerase active site that have been reported to induce higher Exo activity than that of the WT (29), and continued DNA synthesis after incorporating GCV and one additional nucleotide, akin to the DNA extension pattern induced by HCMV Exo mutations (14). Our investigations here distinguish among these hypotheses, revealing a new mechanism of drug resistance, and notably, uncover unexpected results regarding the extension of RNA primers by HCMV Pol.

## RESULTS

### No meaningful effects at the initial step of GCV incorporation.

**FIG 1:**
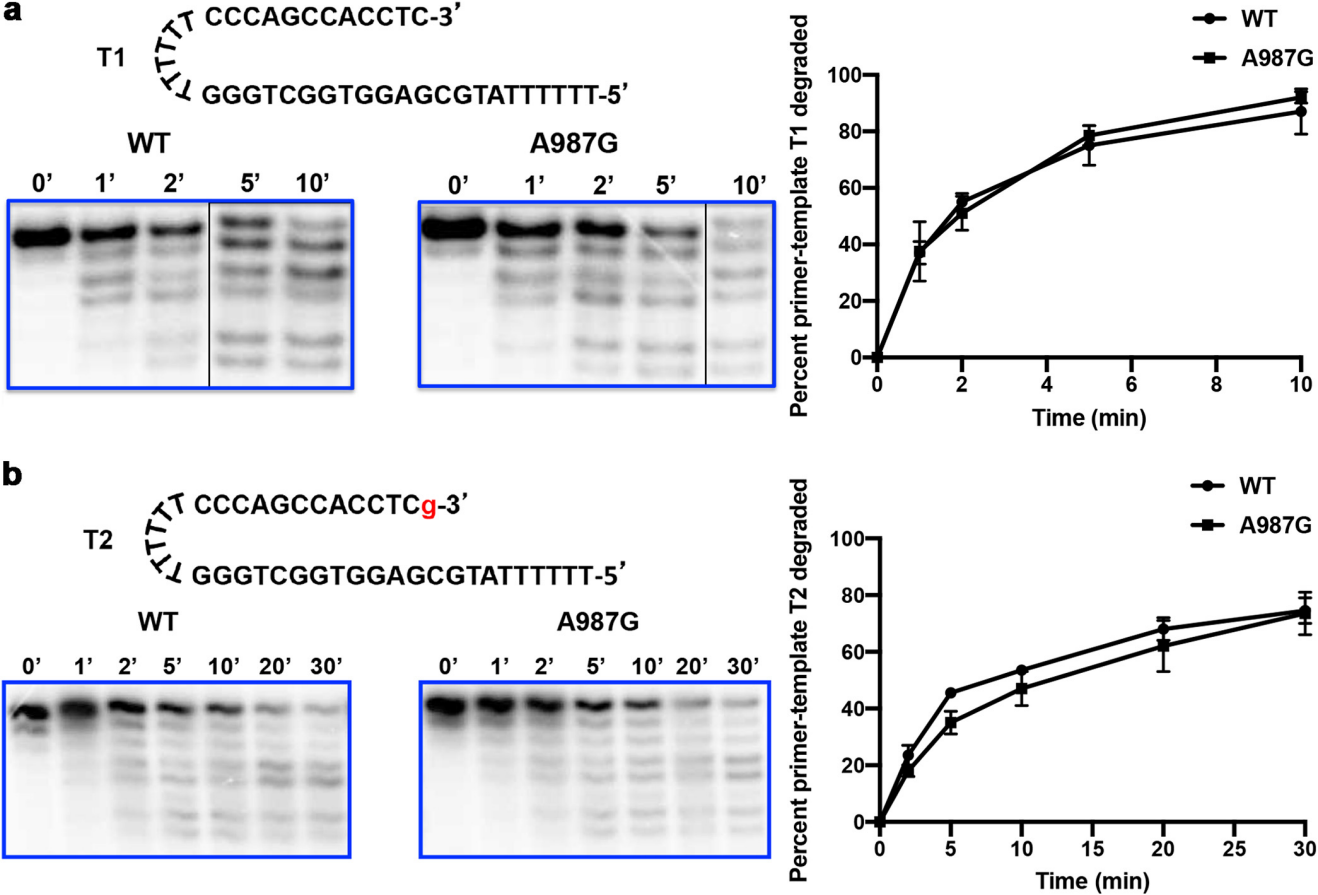
Similar degradation of dC-terminated primer-template T1 (a) and GCV-terminated primer-template T2 (b) by WT Pol and A987G Pol. 5′-Radiolabeled primer-template T1 or T2 (sequences above the gel images in panels a and b, respectively; g, GCV) was incubated with WT Pol or A987G Pol (indicated above each gel image) in the presence of UL44 and absence of dNTPs at 37°C for the times indicated above each lane in the gel image. The reactions were analyzed by polyacrylamide gel electrophoresis and autoradiography (gel images in panels a and b) as well as a phosphorimager to quantify the percentage of starting primer-template that was degraded (graphs on right of panels a and b). Error bars indicate standard errors of the means (SEM) from two independent replicates. The thin black vertical lines in the gel images in panel a indicate where empty lanes were removed from the original images.

To determine how the Pol substitution A987G confers GCV resistance, we purified WT HCMV Pol and A987G Pol as glutathione *S*-transferase (GST) fusion proteins from recombinant baculovirus-infected insect cells (14). We first tested whether the mutant enzyme is impaired for its ability to bind and incorporate GCV-TP into DNA. We used a steady-state enzyme kinetics approach under Michaelis-Menten conditions (11, 14, 28, 30) (see Fig. S1 in the supplemental material), measuring apparent *K_m_* and *k*_cat_ values for incorporation of GCV-TP or dGTP into a 40-mer hairpin primer-template T1 (Fig. 1a), radiolabeled on its 5′ end. We also measured apparent *K_i_* values for GCV-TP inhibition of dGTP incorporation using the same primer-template. UL44, the presumptive HCMV polymerase processivity subunit, was omitted to reduce the contribution of dissociation of polymerase from primer-template to the rate of incorporation.

10.1128/mBio.03492-20.1 FIG S1 Saturable rates of incorporation of dGTP (top) and GCV-TP (bottom). HCMV Pol A987G was incubated at 37°C with an excess of primer-template T1 (Fig. 1a) and various concentrations of dGTP for 5 min (top) or with various concentrations of GCV-TP for 12 min (bottom) as indicated on the *x* axes. The products were analyzed by polyacrylamide gel electrophoresis, and the percentages of incorporated substrates were assessed using a phosphorimager to quantify the single-nucleotide extension product relative to T1 in each lane. The values were converted to rates, and the data were fit to the Michaelis-Menten equation. Error bars indicate SEM from two independent replicates; *R*^2^ values are presented under the curves. Download FIG S1, TIF file, 0.1 MB. Copyright © 2021 Chen et al. 2021 Chen et al. This content is distributed under the terms of the Creative Commons Attribution 4.0 International license.

A987G Pol exhibited apparent *K_m_* values for GCV-TP and dGTP, an apparent *k*_cat_ value for incorporation of GCV-TP, and an apparent *K_i_* for GCV-TP inhibition of dGTP incorporation that were well within 2-fold of those of WT Pol (Table 1). The mutant Pol did exhibit a 2-fold lower apparent *k*_cat_ value for incorporation of dGTP relative to WT Pol, but that would not explain its GCV resistance, as, if anything, reduced incorporation of dGTP would be expected to increase GCV susceptibility. Thus, we found no evidence that the A987G substitution confers GCV resistance at the step of incorporation of GCV-TP into primer-template.

**TABLE 1:** Apparent kinetic constants^*a*^ for incorporation of dGTP and GCV-TP into primer-template T1 by WT Pol and A987G Pol

Name	GCV-TP			dGTP	
	*K_m_*, μM	*k*_cat_, min^−1^	*K_i_*, μM	*K_m_*, μM	*k*_cat_, min^−1^
WT^*b*^	5.5 ± 1.2	1.7 ± 0.17	3.4 ± 1.4	0.44 ± 0.05	22 ± 0.70
987G	5.8 ± 1.5	2.2 ± 0.18	5.1 ± 1.3	0.34 ± 0.09	11 ± 0.67

a Values ± SEM generated from two independent replicates. Apparent *K*_m_ values were determined by fitting data points to the Michaelis-Menten equation using GraphPad Prism (version 6). Apparent *k*_cat_ values were determined by dividing apparent *V*_max_ values by the enzyme concentrations. Apparent *K_i_* values were determined by fitting data to a competitive inhibition model using GraphPad Prism (version 6).

b Apparent kinetic constants for the WT are from references 14 and 59; A987G was analyzed in the same experiments but not reported at the time.

### No increased excision of incorporated GCV.

We next investigated whether the A987G substitution increases the ability of Pol’s 3′–5′ Exo to excise incorporated GCV. Incubation of WT Pol or A987G Pol with either radiolabeled synthetic primer-template T1 terminated with dC (Fig. 1a) or primer-template T2 terminated with GCV (Fig. 1b) in the absence of deoxynucleoside triphosphates (dNTPs) and the presence of the HCMV DNA polymerase accessory subunit UL44 resulted in very similar rates of degradation by both enzymes (Fig. 1). We conclude that the Pol mutation A987G does not confer resistance by increasing excision of incorporated GCV.

### Continued extension after incorporation of GCV-TP.

**FIG 2:**
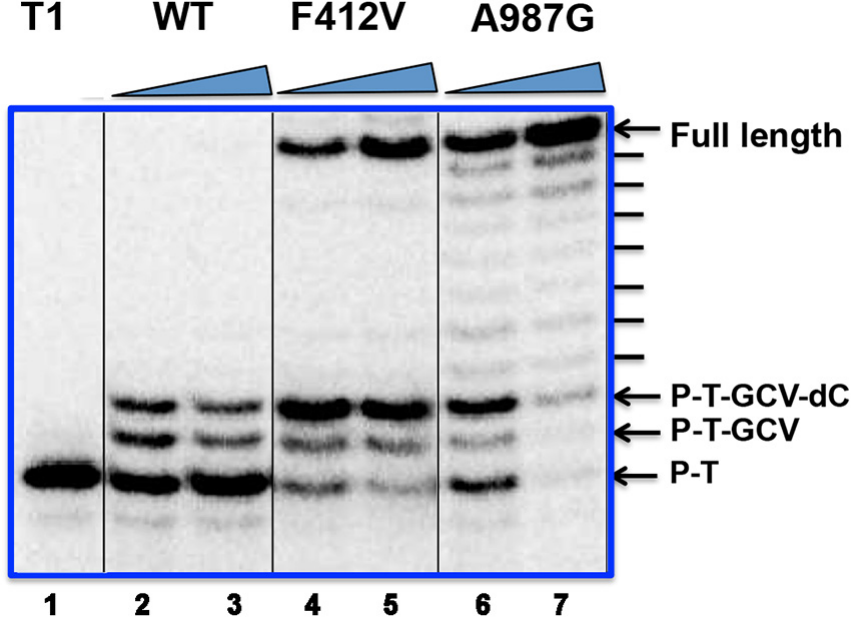
Limited DNA extension by WT Pol and full-length extension by A987G Pol following GCV-TP incorporation. Radiolabeled primer-template T1 (Fig. 1a) was incubated with GCV-TP, dATP, dCTP, dTTP, and each of the indicated Pols (7.2 nM for lanes 2, 4, and 6; 8.4 nM for lanes 3, 5, and 7) in the presence of UL44 at 37°C for 30 min, and the products were analyzed alongside untreated T1 by polyacrylamide gel electrophoresis and autoradiography. Leftmost lane, untreated radiolabeled T1. For the remaining lanes, enzymes used are indicated at the top of the panel, and the wedge indicates increasing concentrations of each enzyme. The arrows to the right of the panel indicate the major species observed. P-T, unmodified primer-template T1; P-T-GCV, T1 with GCV added; P-T-GCV-dC, T1 with GCV and dC added as well as full-length product. The dashes to the right of the panel indicate minor products. The thin black vertical lines in the gel image indicate where lanes containing reactions performed at lower polymerase concentrations and/or using Exo-deficient mutants other than F412V were removed to reduce the size of the image.

We then asked if the A987G substitution allows HCMV Pol to continue DNA synthesis after incorporation of GCV-TP and the subsequent nucleotide at the N + 1 position, akin to the DNA extension pattern induced by GCV^r^ Exo mutations (14). To this end, WT Pol, A987G Pol, and, as a positive control, the HCMV Exo mutant F412V that we characterized previously (14) were each incubated with radiolabeled primer-template T1 (Fig. 1a) in the presence of UL44, GCV-TP, dATP, dCTP, and dTTP (dGTP was omitted). As expected, WT Pol efficiently terminated DNA synthesis after incorporating GCV-TP plus one additional nucleotide (in this case, dCTP) (Fig. 2). Consistent with our previous study (14), Exo mutant F412V was able to continue DNA synthesis to the end of the template after incorporating GCV-TP and dCTP, despite ∼50% termination at the N + 1 position (Fig. 2). Interestingly, after A987G Pol incorporated GCV-TP into primer-template T1, there was less termination following incorporation of dCTP into the N + 1 position, with most DNA synthesis continuing to the end of the template, particularly at the higher enzyme concentration (Fig. 2). Similar to previous results with Exo-deficient mutants (14), the full-length products generated by A987G Pol were not the result of misincorporation of dATP, dCTP, or dTTP opposite dC in the template, because there was no DNA extension in the absence of GCV-TP, and full-length products synthesized in the presence of GCV-TP exhibited altered electrophoretic mobility relative to those synthesized in the presence of dGTP (Fig. S2). These results support the hypothesis that the A987G substitution confers resistance by permitting the enzyme to continue DNA synthesis following incorporation of GCV plus the next nucleotide.

10.1128/mBio.03492-20.2 FIG S2 Full-length product requires GCV-TP and migrates aberrantly. Radiolabeled primer-template T1 (Fig. 1a) was loaded alone on a polyacrylamide gel as a marker (leftmost lane); incubated with A987G Pol and UL44 plus dCTP, dATP, and dTTP, which resulted in no detectable products (second lane from left); incubated with A987G Pol and UL44 plus dGTP, dCTP, dATP, and dTTP (second lane from right); or incubated with A987G Pol and UL44 plus GCV-TP, dCTP, dATP, and dTTP (rightmost lane). The arrows to the right of the panel indicate the major species observed. P-T, unmodified primer-template T1; P-T-GCV, T1 with GCV added; P-T-GCV-dC, T1 with GCV and dC added, and full-length products synthesized with dGTP but without GCV-TP (without GCV) or with GCV-TP but not dGTP (with GCV). The dashes to the right of the panel indicate minor products. The thin vertical black lines indicate where an empty lane and lanes with redundant controls were removed from this image to reduce its size and complexity. Download FIG S2, TIF file, 0.9 MB. Copyright © 2021 Chen et al. 2021 Chen et al. This content is distributed under the terms of the Creative Commons Attribution 4.0 International license.

### A987G Pol still idles.

**FIG 3:**
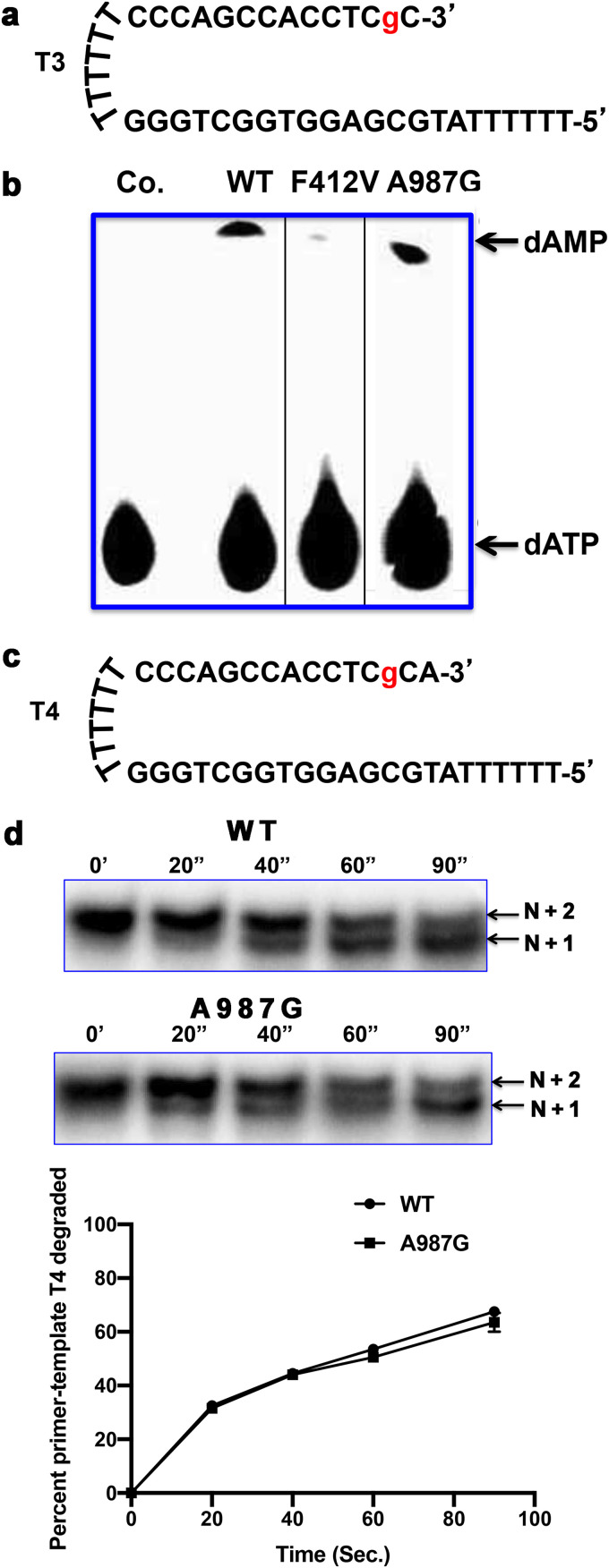
Similar idling and exonuclease activity of WT Pol and A987G Pol on primer-templates containing internally incorporated GCV. (a) The sequence of N + 1 primer-template T3; g, GCV. (b) T3 was incubated with [α-^32^P]-labeled dATP either in the absence of enzyme (Co.) or using the enzymes indicated at the top of the panel in the presence of UL44 at 37°C for 10 min, and the products were analyzed by TLC and autoradiography. The positions of dATP and dAMP were determined by visualizing unlabeled standards under UV light and are indicated by arrows to the right of the panel. The thin black vertical lines indicate where lanes from reactions with Exo-deficient mutants other than F412V were removed from the image to reduce its size. (c) The sequence of N + 2 primer-template T4; g, GCV. (d) T4 was incubated with WT Pol and A987G Pol in the presence of UL44 and absence of dNTPs for the times indicated above each panel. The products were analyzed by polyacrylamide gel electrophoresis and autoradiography (top) or a phosphorimager (bottom), which was used to quantify the percentage of starting primer-template that was degraded. Error bars indicate SEM from two independent replicates. The arrows to the right of the autoradiogram indicate the positions of T4 (N + 2) and the product (N + 1) generated by removal of a single nucleotide from T4.

Following incorporation of GCV-TP and the subsequent nucleotide, WT Pol repeatedly adds the incoming dNTP and then rapidly removes it to generate dNMP (idling), thereby terminating extension at the N + 1 position (14). In contrast, Exo mutants exhibit little or no idling, so they overcome chain termination and continue synthesis (14). To test whether A987G Pol eliminates idling, we incubated WT Pol, A987G Pol, and, as a control, Exo mutant F412V Pol with a synthetic primer-template T3 terminated with GCV plus dC (Fig. 3a) and assayed for generation of labeled dAMP in the presence of [α-^32^P]dATP using thin-layer chromatography (TLC). In this assay, both WT Pol and A987G Pol converted similar amounts of dATP to dAMP (both ∼10%), and substantially more than F412V Pol converted (∼0.7%) (Fig. 3b), indicating that the A987G substitution did not eliminate idling. To follow up this finding, we examined whether WT Pol and A987G Pol could efficiently degrade a radiolabeled primer-template, T4 (Fig. 3c), terminated with GCV plus dC and, in the N + 2 position, dA. Consistent with the results of the idling assay in the absence of dNTPs, both WT (as previously observed [14]) and A987G Pols rapidly degraded primer-template T4 at indistinguishable rates (Fig. 3d), generating N + 1 primer-template without further degradation (Fig. 3d). As before (14), F412V Pol was substantially impaired for degrading the N + 2 primer-template T4 (Fig. S3). Taken together, these results indicate that the A987G substitution does not eliminate idling, and that A987G Pol and the Exo mutant F412V Pol use different mechanisms to enable continued DNA synthesis following incorporation of GCV-TP into DNA.

10.1128/mBio.03492-20.3 FIG S3 Exo mutant F412V Pol is highly impaired for its degradation of T4 relative to WT Pol. Radiolabeled primer-template T4 (Fig. 3C) was incubated with WT Pol or F412V Pol in the presence of UL44 and absence of dNTPs for the various times indicated above each lane of the gel image. The products were analyzed by polyacrylamide gel electrophoresis and autoradiography. The thin black vertical lines indicate where empty lanes were removed from the original image. Download FIG S3, TIF file, 0.1 MB. Copyright © 2021 Chen et al. 2021 Chen et al. This content is distributed under the terms of the Creative Commons Attribution 4.0 International license.

### More rapid extension of GCV-containing N + 1 and N + 2 primers.

**FIG 4:**
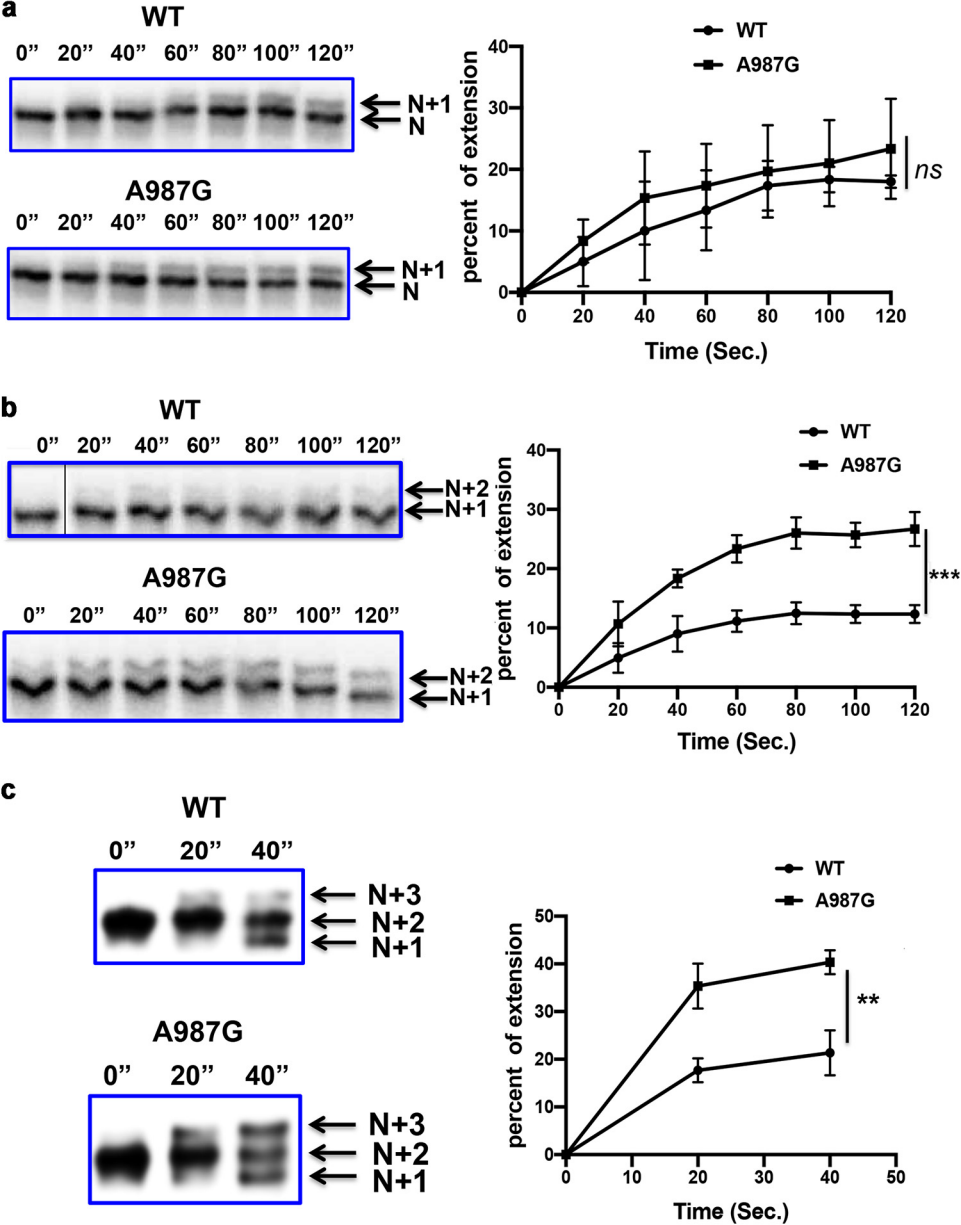
Single-nucleotide incorporation by WT Pol and A987G Pol on GCV-containing primer-templates. Radiolabeled primer-template T2 terminating with GCV (Fig. 1b) plus dCTP (a), N + 1 primer-template T3 (Fig. 3a) plus dATP (b), or N + 2 primer-template T4 (Fig. 3c) plus dTTP (c) was incubated with WT Pol or A987G Pol in the presence of UL44 at 37°C for the times indicated above each lane of the gel images. The products were analyzed by polyacrylamide gel electrophoresis and autoradiography. The positions of the starting primer-template and each product are indicated by arrows (right side of the gel image in each panel). The thin vertical black line in the image in panel b indicates where an empty lane was removed from the original image. The percentages of products that were larger (by one nucleotide) than their corresponding primer-templates in each gel lane were quantified using a phosphorimager and are plotted against incubation time (in panel c, only the products generated within 40 s were analyzed due to the rapid degradation of the T4 primer-template). Error bars shown in the plot of each panel indicate standard deviations based on three independent experiments. *P* values were obtained using repeated-measures two-way analysis of variance in GraphPad Prism 8. *ns*, not significant; **, *P = *0.0014; ***, *P = *0.0029.

We hypothesized that the A987G substitution increases the enzyme’s rate of extension of primers containing GCV, allowing the polymerase to overcome GCV-induced chain termination and continue DNA synthesis. To test this hypothesis, we first analyzed the rates of dCTP incorporation by WT and mutant Pols on the N primer-template T2 terminated with GCV (Fig. 1b). The incubation of radiolabeled primer-template T2 with WT Pol or A987G Pol in the presence of UL44 and dCTP (no other dNTPs) resulted in similar rates of formation of N + 1 product (Fig. 4a and Table 2). Next, we examined dATP incorporation on the N + 1 primer-template T3 terminated with GCV plus dC (Fig. 3a) to assess incorporation of the second nucleotide (N + 2). A987G Pol (with UL44) extended the N + 1 primer-template more rapidly than did WT (Fig. 4b and Table 2). Finally, when we incubated the enzymes with radiolabeled N + 2 primer-template T4 terminated with GCV-dC-dA (Fig. 3c) in the presence of UL44 and dTTP, A987G Pol again generated N + 3 products more rapidly than did WT Pol (Fig. 4c and Table 2; extension plateaued after 20 s of incubation, accompanied by rapid Exo degradation of the starting N + 2 primer-template and N + 3 products).

**TABLE 2:** Comparison of the rates^*a*^ for extension and degradation of each primer-template by WT Pol and A987G Pol

P-T	Nucleoside	Enzyme (nM)	Extension^*b*^(nM/min)		Degradation^*b*^ (nM/min)	
			WT	A987G	WT	A987G
T1	GCV-TP	30	48 ± 2	53 ± 3	30 ± 5	31 ± 3
T2 (N)	dCTP	10	30 ± 5	38 ± 5	20 ± 4	15 ± 5
T3 (N + 1)	dATP	10	6 ± 1	14 ± 2	ND^*c*^	ND^*c*^
T4 (N + 2)	dTTP	30	36 ± 1	72 ± 4	59 ± 3	57 ± 2

a The extension rates for incorporation of GCV-TP or the relevant dNTP indicated in each row and the degradation rates on each primer-template were determined by plotting the amount of extended or degraded primer-templates versus time. P-T, primer-template.

b Errors are SEM generated from two independent replicates.

c ND, not detectable.

We compared extension and degradation rates on each primer-template by WT Pol and A987G Pol (Table 2). (Because the rates of extension and degradation varied among different primer-templates, different concentrations of Pol were used [Table 2] to permit sufficient detection of product while achieving linear rates). WT Pol and A987G Pol extended primer-template T1 by incorporating GCV-TP into the N position at similar rates (Table 2, Fig. S4), consistent with their similar apparent *k*_cat_ values (Table 1). These rates of GCV-TP incorporation were relatively low compared with extension using dGTP (Table 1) and only ∼1.7-fold higher than the degradation rates on the same primer-template. On primer-template T2, which contains GCV in the N position, the two enzymes again exhibited similar rates of incorporation of dCTP at the N + 1 position that were just slightly higher than the degradation rates. On primer-template T3, no degradation could be detected (as previously observed [14]). Here, although the rates of extension were low for both enzymes, A987G Pol exhibited an ∼2-fold higher extension rate than the WT in incorporating dATP at the N + 2 position. Once incorporated, WT enzyme more rapidly removes that nucleotide than extends it to the N + 3 position, as seen using primer-template T4 (Fig. 4c). In contrast, A987G not only more rapidly generates primer-template terminated at the N + 2 position than does WT but also more rapidly extends it to the N + 3 position, with the rate of extension being ∼2-fold greater than that of the WT. Importantly, that rate of extension is greater than the rate of degradation of the N + 2 primer-template (Table 2), permitting DNA synthesis to continue. These analyses support a model for how A987G Pol overcomes GCV-induced chain termination, which we summarize below in Discussion.

10.1128/mBio.03492-20.4 FIG S4 WT Pol and A987G Pol incorporate GCV-TP into primer-template T1 at similar rates. Radiolabeled T1 (Fig. 1a) was incubated with WT Pol or A987G Pol in the presence of UL44 and GCV-TP (no other nucleotides) for the times indicated on the *x* axis. The products were analyzed by polyacrylamide gel electrophoresis, and the percentage of products with incorporated GCV-TP was assessed using phosphorimager to quantify the one nucleotide larger band relative to T1 at each time point. The error bars indicate SEM from two independent replicates. Download FIG S4, TIF file, 0.1 MB. Copyright © 2021 Chen et al. 2021 Chen et al. This content is distributed under the terms of the Creative Commons Attribution 4.0 International license.

### Utilization of RNA primers.

In the nuclear magnetic resonance (NMR) structure of a 10-bp oligonucleotide containing GCV, the internal GCV moieties induce distortion in the DNA backbone so that it locally resembles A-DNA (31). Given that an RNA primer bound to DNA template results in an A-form duplex (32), we hypothesized that the A987G mutant Pol can extend RNA primers relatively efficiently. To our knowledge, HCMV Pol has not previously been tested for its ability to extend RNA primers.

**FIG 5:**
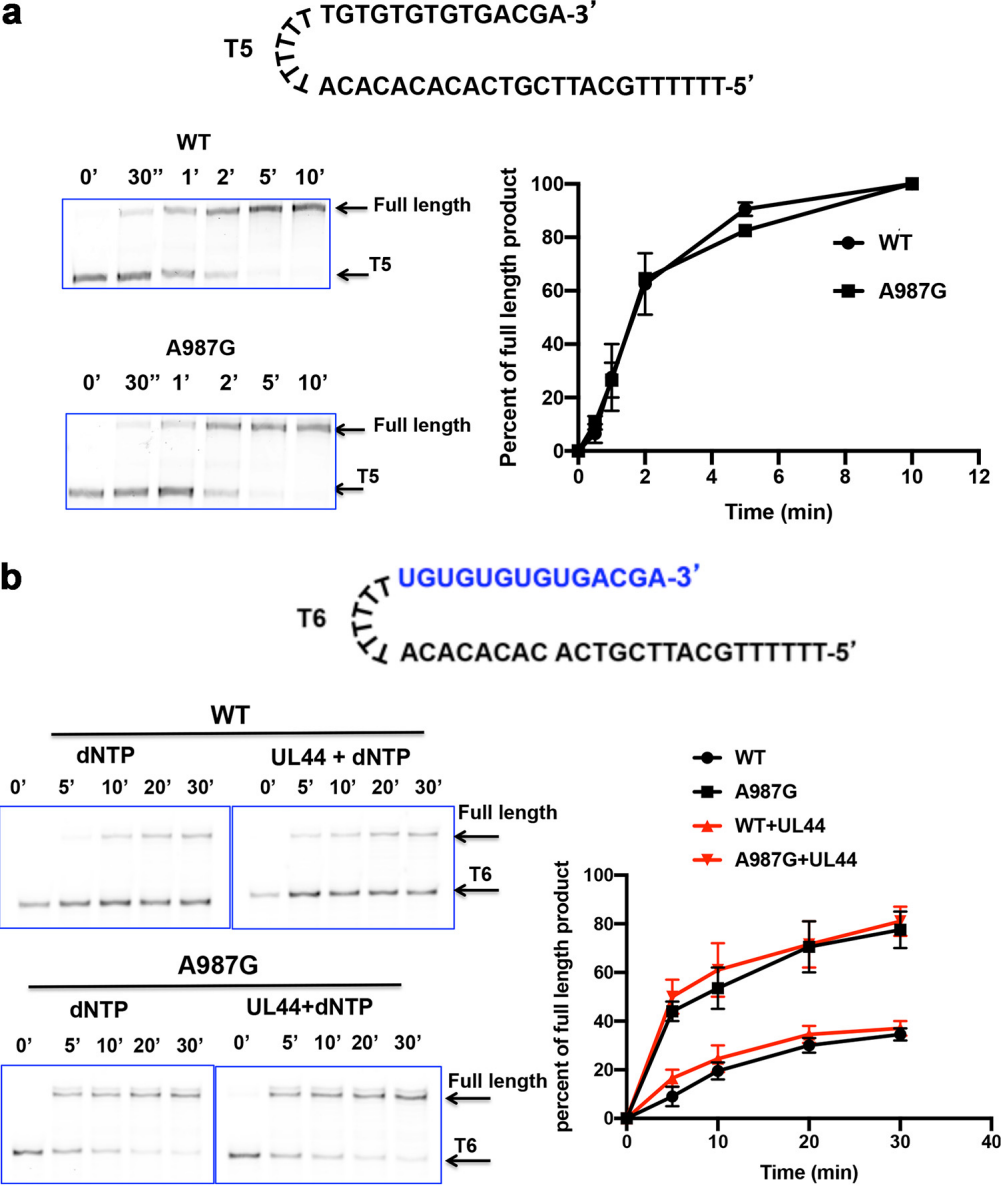
Extension of DNA and RNA primers by WT Pol and A987G Pol. (a, top) The sequence of FAM-labeled DNA primer-template T5. (Left) T5 was incubated with each of the indicated Pols and dNTPs at 37°C for the times indicated on the top of each lane of the gel image. The products were analyzed by polyacrylamide gel electrophoresis and fluorescent imaging. The arrows to the right side of the gel images indicate the positions of full-length products and T5. (Right) The percentages of full-length products were quantified using ImageJ and were plotted against incubation time. Error bars indicate SEM based on two independent replicates. (b, top) The sequence of 6-FAM-labeled primer-template T6; the RNA portion is in blue. T6 was incubated with each of the indicated Pols and dNTPs in the presence or absence of UL44 at 37°C for the times indicated on the top of each lane of the gel image on the left. The full-length DNA products were analyzed, quantified, and plotted against the incubation time on the right as described for panel a. The positions of full-length DNA product and primer-template T6 are indicated by arrows to the right of the gel image, and error bars in the plot indicate SEM from two independent replicates.

To test this hypothesis, we used two 5′ fluorescently labeled hairpin primer-templates, here named T5 and T6 (previously designated S1 and S2 [33]), with the same template sequence but with T5 containing a DNA primer (Fig. 5a) and T6 containing the corresponding RNA primer (Fig. 5b). As a control, we initially tested WT HSV-1 Pol using these two primer-templates. Despite reports that HSV-1 Pol extends RNA primers inefficiently (25, 34), we found that it could extend the RNA primer in T6 effectively, converting all primer-template to full-length products, albeit less rapidly than its extension of the DNA primer in T5 (Fig. S5).

10.1128/mBio.03492-20.5 FIG S5 Extension of DNA or RNA primers by WT HSV-1 Pol. FAM-labeled DNA primer-template T5 (top) or FAM-labeled primer-template T6 (bottom), containing an RNA primer (RNA portion in blue), was incubated with HSV-1 Pol and dNTPs at 37°C for the times indicated above each lane of the gel image. The products were analyzed by polyacrylamide gel electrophoresis and fluorescent imaging. Download FIG S5, TIF file, 0.5 MB. Copyright © 2021 Chen et al. 2021 Chen et al. This content is distributed under the terms of the Creative Commons Attribution 4.0 International license.

We then tested WT Pol and A987G Pol. Both enzymes extended the DNA primer in T5 at similar rates, with nearly half of the primer extended within 30 s (Fig. 5a). Both enzymes also extended the RNA primer in T6 to full-length products (Fig. 5b). In line with our hypothesis, A987G Pol extended RNA primers more efficiently than the WT, with 60% of RNA primer converted to full-length products after 5 min of incubation, while WT Pol only converted 10% of RNA primers to full-length products within the same time (Fig. 5b). The addition of UL44 had little, if any, effect on the ability of the enzymes to extend an RNA primer (Fig. 5b). Thus, HCMV Pol can utilize and extend RNA primers, and the A987G mutation increases that activity.

## DISCUSSION

**FIG 6:**
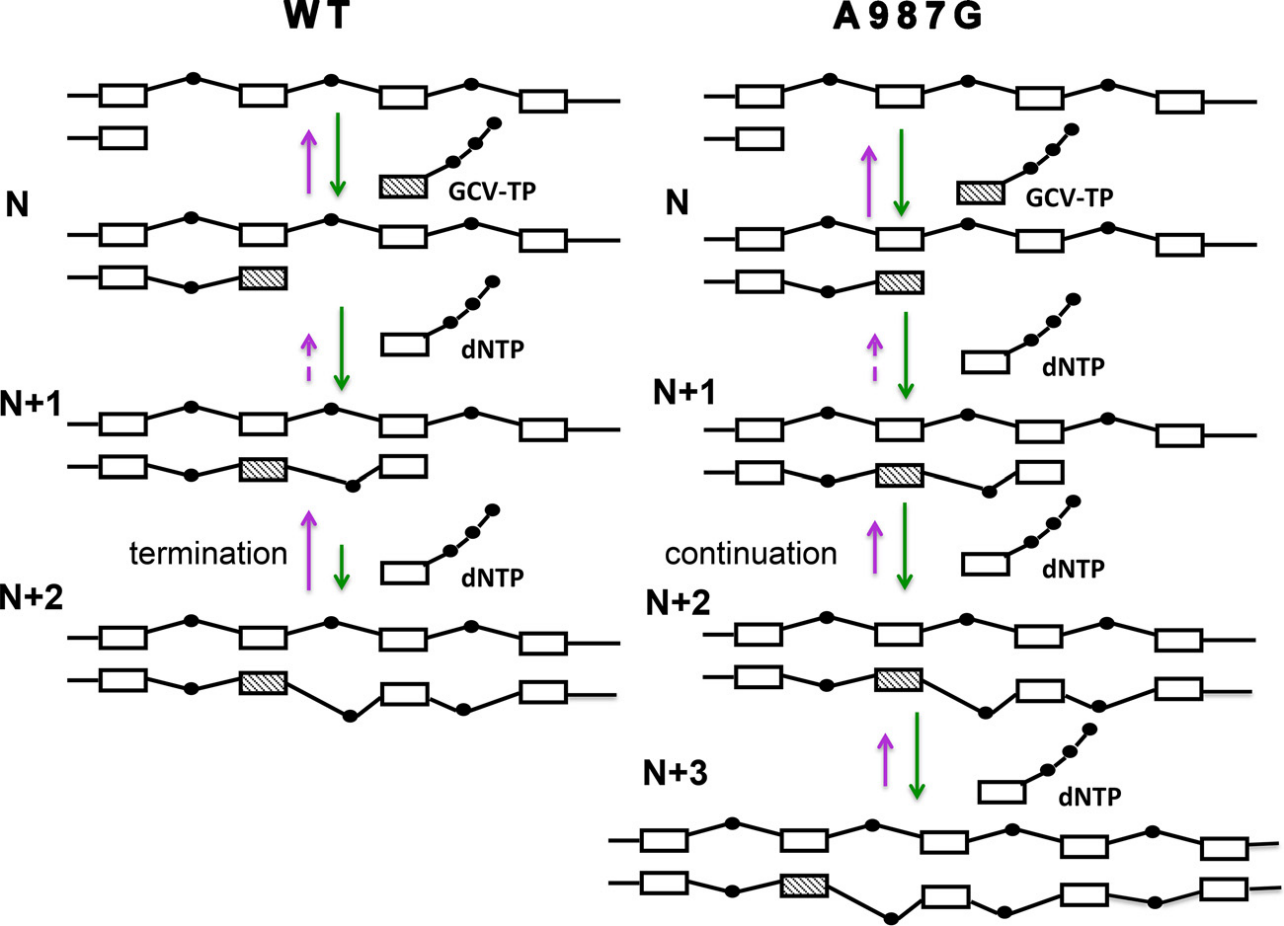
Model for chain termination by WT Pol (left) and chain elongation by A987G Pol (right) after incorporating GCV and one additional nucleotide. Each open box and the black dot that follows in the template strand represented at the top indicate deoxynucleoside monophosphates within DNA. The primer strand grows from left to right and incorporates GCV-TP (hatched box with a line of three black dots) to form the N primer-template and subsequently natural dNTPs (open box with a line of three black dots) at each position (N + 1, N + 2, and N + 3), indicated at the left side of corresponding primer-template. The asymmetric lines connecting GCV and the subsequent two nucleotides at N + 1 and N + 2 indicate the distortion in the sugar-phosphate backbone induced by GCV (57). Turquoise vertical arrows pointing down represent incorporation, and the magenta arrows pointing up represent excision. The relative lengths of turquoise and magenta arrows indicate relative rates for extension and degradation of each primer template. The short dashed magenta lines represent the undetectable degradation of N + 1 primer template. See the text for a discussion.

Mutations in polymerase genes that confer resistance to nucleoside analogs usually do so by reducing binding and/or incorporation of drug TPs or by increasing excision of incorporated drugs (3). In contrast, HCMV Pol Exo mutants confer resistance to GCV by preventing idling, permitting the extension of GCV-containing primers (14). Here, we found the HCMV Pol mutant A987G, with a substitution in a conserved motif (region V) of the thumb domain, adopts an entirely new mechanism to confer resistance to GCV. As summarized in Fig. 6, like WT enzyme, this mutant can incorporate GCV-TP plus the subsequent nucleotide (N + 1 position). Like WT enzyme, its Exo activity does not detectably degrade this primer-template but idles due to high Exo activity on the N + 2 extension product. However, unlike WT enzyme, A987G Pol can escape idling and thus overcome chain termination at the N + 1 position by extending both the N + 1 and N + 2 drug-containing primer-templates more rapidly than its Exo can degrade them.

**FIG 7:**
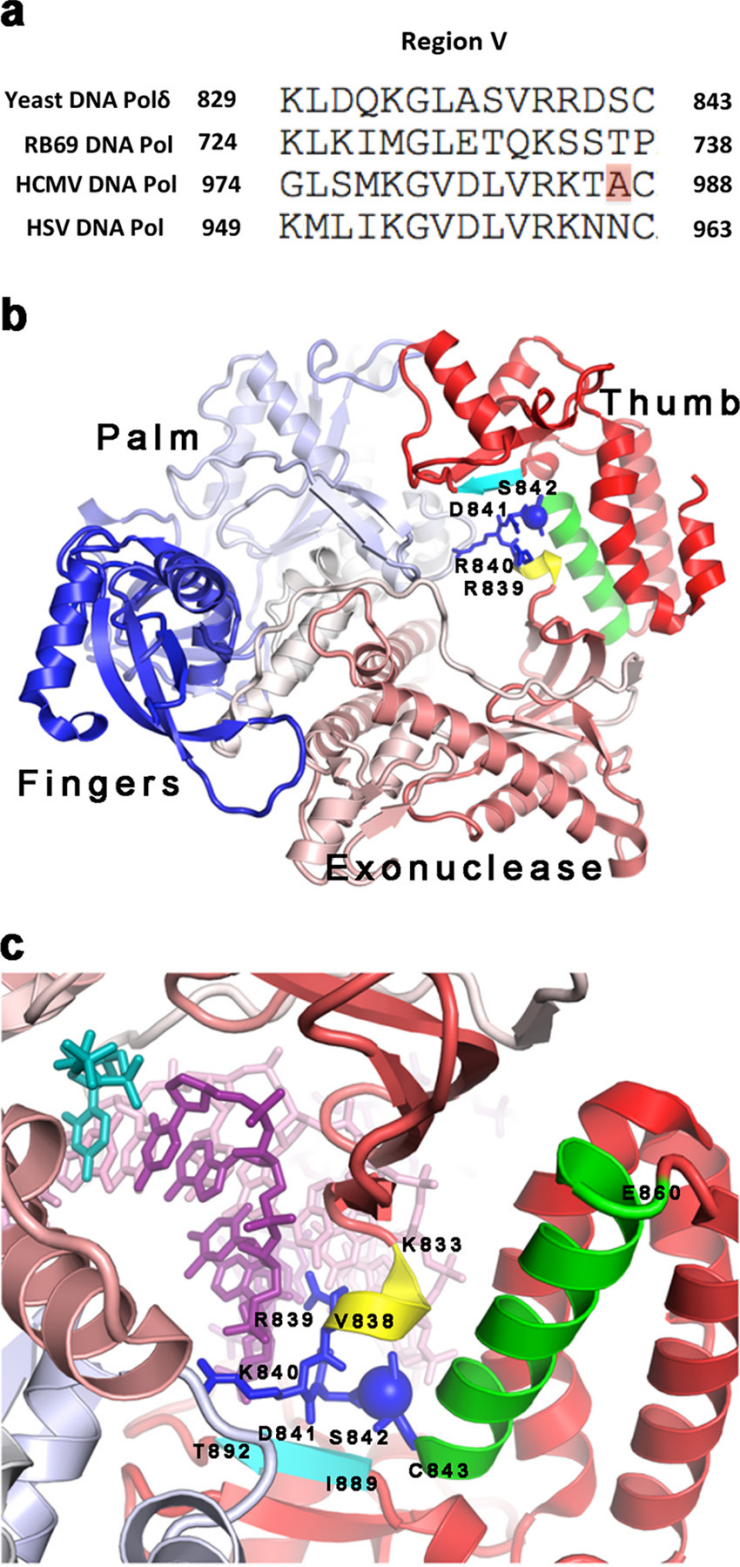
Location and contacts of the residue corresponding to HCMV residue A987 in the polymerase active site of yeast DNA polymerase δ bound to primer-template and dNTP (PDB entry 3IAY). (a) Amino acid sequence alignment of conserved region V of family B DNA polymerases from yeast DNA polymerase δ (Polδ), bacteriophage RB69 DNA Pol, HCMV DNA Pol, and HSV-1 DNA Pol. The position of HCMV Pol residue A987 is highlighted in red. (b) The Pol δ fingers, palm, most of the thumb and exonuclease domains are shown in dark blue, white, red, and light orange, respectively. The conserved RRxx motif (yeast Pol δ R839, R840, D841, S842), corresponding to residues R984, K985, T986, and A987 of HCMV Pol, is shown as a dark blue stick model. S842, corresponding to HCMV Pol reside A987, is highlighted as a dark blue sphere. An adjacent α-helix, β-turn, and β-strand are shown in green, yellow, and cyan, respectively. DNA and the incoming nucleotide in the structure are omitted for clarity. (c) Close-up view of the contacts of residue S842 corresponding to HCMV Pol A987. The blue stick model represents residues 839 to 842 with residue 842 shown as a blue sphere. Residues I889 to T892 in the Pol δ sequence, which form a β-strand, and residues C843 to E860, which form a long α-helix, are colored in cyan and green, respectively. Residues K833 to V838, corresponding to the site of an HCMV deletion that confers drug resistance, form a β-turn and are highlighted in yellow. The DNA primer strand, template strand, and incoming nucleotide dCTP in the structure are in dark purple, pink, and teal, respectively.

The A987G substitution alters conserved region V of family B DNA polymerases, which includes a sequence motif with two basic residues followed by two less conserved residues (R984, K985, T986, and A987 for WT HCMV) (Fig. 7a). There is currently no published structure of a herpesvirus Pol bound to primer-template. To understand the effects of the A987G mutation on Pol’s activity to extend GCV-containing DNA primers, we examined the structure of the most closely related family B DNA polymerase that has been solved bound to a DNA primer-template (and an incoming nucleotide): yeast (Saccharomyces cerevisiae) DNA polymerase δ (23) (PDB entry 3IAY). In this model, the sequence motif R839, R840, D841, and S842 in region V of the thumb subdomain corresponds to the motif containing residues R984, K985, T986 and A987 in HCMV Pol (Fig. 7b). The two basic residues (yeast R839 and R840) contact the backbone of the primer strand, which is in turn base paired with template (Fig. 7c). The yeast residue corresponding to HCMV A987 (in this case S842) makes no contacts with DNA, but its side chain does help hold these basic residues in place by interacting with a section of alpha helix (C843 to E860) just N-terminal of the basic residues and with a nearby beta strand formed by residues I889 to T892 (Fig. 7c; a roughly similar arrangement is found in the structure of another family B polymerase bound to primer-template and incoming nucleotide, the phage RB69 Pol [22]) (Fig. S6a; PDB entry 1IG9). Replacement of this residue with a glycine (G), which is highly flexible and whose side chain (H) would not interact with other nearby protein segments, would be expected to make the interaction with primer less rigid and, thus, better able to accommodate the changes in the primer backbone due to incorporated GCV (31) or ribonucleoside moieties. The less rigid structure might also account for the slightly lower apparent *k*_cat_ than that of the WT for dGTP incorporation. We note the possibility that local sequence variations might modulate the distortions induced by incorporated GCV, which in turn could affect how efficiently GCV induces chain termination and how effectively the A987G mutation overcomes such termination.

10.1128/mBio.03492-20.6 FIG S6 Residue T737, which corresponds to HCMV Pol residue A987G, in the polymerase active site of phage RB69 Pol bound to primer-template and incoming nucleotide (PDB entry 1IG9) (a) and in the exonuclease active site of phage RB69 Pol in editing mode (PDB entry 1CLQ) (b). (a) Most of the RB69 thumb and exonuclease domains are in red and purple, respectively. The conserved RRxx motif (K734 to T737) is shown as a dark blue stick model, and residue T737, corresponding to HCMV Pol A987G, is highlighted as a sphere. The β-strand (A781 to S784), the long α-helix (P738 to E755), and the β-turn (L730 to Q733) in the vicinity are shown in cyan, green, and yellow, respectively. The DNA primer strand, template strand, and incoming nucleotide are colored in dark purple, pink, and teal, respectively. The two divalent ions bound at the polymerase active site are colored orange. (b) Color coding for the residues in the exonuclease active site of RB69, the DNA primer template, and metal ions is the same as those in the polymerase active site in panel a. The side chains of residues that bind the orange-colored divalent ions are also labeled orange, with the location of residue D114, which corresponds to HCMV Pol residue D301, shown with an arrow. The position of residue F221, which corresponds to HCMV Pol residue F412 and lies at the top of a short α-helix behind the divalent cations, is also shown with an arrow. The dark blue conserved RRxx motif, corresponding to residues R984, K985, T986, and A987 in HCMV Pol moves away from the blue β-strand (A781 to S784). Download FIG S6, TIF file, 2.6 MB. Copyright © 2021 Chen et al. 2021 Chen et al. This content is distributed under the terms of the Creative Commons Attribution 4.0 International license.

To understand the lack of effect of the A987G mutation on Exo activity, we also examined the structure of the family B DNA polymerase from phage RB69 bound to primer-template, in this case with primer in the Exo active site (35) (PDB entry 1CLQ). In this structure, the residues corresponding to HCMV Pol R984 and K985, or any other residues expected to be affected by changes in the residue corresponding to HCMV Pol A987, do not interact with primer (Fig. S6b). This would explain why the HCMV A987G substitution does not affect Exo activity. Thus, these structures provide a rationale for how A987G overcomes GCV-mediated chain termination.

Interestingly, the HCMV equivalent of the helical section (residues K833 to V838 in Fig. 7c), with which the yeast DNA polymerase δ residue corresponding to HCMV Pol A987 interacts, is the site of a deletion in an HCMV mutant (36). This deletion, like A987G, confers resistance to GCV and to the FDA-approved drug cidofovir (CDV), another delayed, nonobligate chain terminator that likely has a mechanism of termination similar to that of GCV (14, 37). We hypothesize that this mutant would, like A987G, exhibit increased rates of extension of GCV-containing primers.

Polymerase mutants from other systems containing mutations in the thumb subdomain (24, 28, 29, 38) have been reported to exhibit decreased polymerase activity relative to their WT counterparts, and these defects can be largely attributed to effects on primer binding. A987G Pol does exhibit a modest decrease in *k*_cat_ for dGTP incorporation, but the A987G substitution does not appear to meaningfully affect replicative fitness of the virus in cell culture (39). As this mutant has arisen in patients (18–20), it is also not highly crippled *in vivo*.

Our findings that WT HCMV Pol can readily extend RNA primers, which, to our knowledge, had not been previously reported, touch on a long-running literature regarding what enzyme extends RNA primers during herpesvirus DNA synthesis (25, 26, 27, 34, 40, 41). In particular, a thorough study from the Kuchta laboratory found that host DNA polymerase α-primase extended primers synthesized by the HSV-1 helicase-primase much more efficiently, i.e., much higher *V*_max_/*K_m_* values, largely due to a much lower *K_m_* for dNTPs than the viral DNA polymerase (25). However, the Kuchta laboratory then elegantly demonstrated that viral proteins alone were sufficient for both leading- and lagging-strand synthesis on DNA minicircle templates and that DNA polymerase α-primase had no effect in this assay (34). These authors attributed the discrepancy in part to differences in template concentrations.

In the present study, we found much less marked differences in the extension of RNA and DNA primers by WT HSV-1 and HCMV Pols. Our studies were performed at high dNTP concentrations, where the Kuchta laboratory also found fairly similar rates of extension of RNA and DNA primers by HSV-1 DNA polymerase (25). As dNTP concentrations in HSV- and HCMV-infected cells are relatively high (16, 42), we think it likely that the viral DNA polymerases can efficiently extend RNA primers. During initiation of HCMV DNA synthesis, the viral polymerase might use transcripts generated by host RNA polymerase II that arise from or near the origin of replication (43, 44) as primers. During lagging strand synthesis, it would extend primers laid down by the viral helicase-primase. As the HCMV Pol A987G substitution increases the ability of the enzyme to utilize RNA primers, that might promote fitness of the mutant in the face of its reduced *k*_cat_ for dNTP incorporation.

Our results are relevant to other antiviral and anticancer nucleoside analogs that are nonobligate chain terminators. Some of them act in a delayed fashion, like GCV, while others terminate DNA synthesis at the site of their incorporation. As mentioned above, the A987G substitution, but also several other alterations in the polymerase domain of HCMV Pol, confer resistance to CDV (37, 45). A more orally available prodrug of CDV, brincidofovir, is being developed to treat other DNA virus infections (46, 47). Enhanced extension of CDV-containing primers by A987G, and possibly other polymerase domain mutants, seems likely to account for its resistance to CDV. This hypothesis is currently under investigation. Similar mutations could emerge in other DNA viruses encoding family B polymerases. It is also conceivable that mutations enhancing elongation of drug-containing primers by host DNA polymerases affect the use of GCV in suicide therapies or contribute to resistance to nonobligate chain-terminating anticancer drugs, including ones with chain termination mechanisms that differ from that of GCV (e.g., gemcitabine [48]). In all these cases, as previously discussed (14), replicated DNA would contain internally incorporated nucleoside analogues that might increase mutation frequencies or strongly impair DNA synthesis when the replication machinery attempts to copy the DNA strands that contain drug. As a number of GCV^r^ Exo-deficient HCMV isolates and the A987G mutant are fit enough to replicate and cause disease in patients (13, 18–20), we hypothesize that DNA repair and/or recombination mechanisms may remove drug moieties from DNA to permit high rates of DNA synthesis without excessively high mutation frequencies.

We further speculate that mutations that enhance elongation of drug-containing primer strands contribute to resistance to approved and investigational nonobligate chain terminators of reverse transcription or RNA synthesis, such as certain anti-hepatitis B drugs (e.g., entecavir [49]), anti-human immunodeficiency virus drugs (e.g., islatravir [50]), anti-hepatitis C drugs (e.g., sofosbuvir [51]), and, of particular current interest, the anti-coronavirus drug remdesivir (52–56). In these cases, mechanisms other than exonuclease-mediated idling, such as slowed translocation, pyrophosphate-mediated excision, and/or polymerase backtracking, would be overcome by enhanced extension to overcome chain termination. Whether resistance would then require repair or recombination mechanisms to remove incorporated drug and whether such mechanisms would be available remain open questions.

## MATERIALS AND METHODS

### Construction of recombinant baculoviruses.

Recombinant baculoviruses used to express WT Pol and A987G Pol were constructed using bacmids and methods described previously (14). Primers used for site-directed mutagenesis of plasmid pGST-WT Pol to generate the plasmid for glutathione *S*-transferase (GST)-tagged HCMV Pol mutant A987G were purchased from Integrated DNA Technologies: CTGGTGCGCAAGACGGGCTGCGAGT (forward) and CTTGACGAACTCGCAGCCCGTCTTGC (reverse).

### Protein purification.

WT Pol and A987G Pol, expressed as GST fusion proteins, were overexpressed in insect cells and purified using affinity chromatographic methods described previously (14). WT HSV-1 Pol was expressed as a 6×His-tagged protein and purified using affinity chromatography as described previously (33).

### Oligonucleotides.

Primer template T1 and 6-carboxyfluorescein (FAM)-labeled primer-template T5 and T6 were purchased from Integrated DNA Technologies. Primer-templates T2, T3, and T4 were synthesized by ChemGenes using GCV phosphoramidite prepared as described by Marshalko et al. (57). Sequences were confirmed in a synthesis report, and were validated by molecular mass determined by ESI mass spectrometry, and by step-wise incorporation of dNTPs by HCMV Pol. Purity was established by capillary electrophoresis.

### Enzyme assays.

Six different assays were performed: polymerase assays to measure apparent kinetic parameters (*K_m_*, *k*_cat_, and *K_i_*), exonuclease assays, assays of full-length extension in the presence of GCV-TP, idling assays, single-nucleotide extension on GCV-containing primer-templates, and assays of full-length extension on FAM-labeled primer-templates using either RNA or DNA primers. Unless otherwise noted, all reactions with HCMV Pols were performed in 10-μl volumes at 37°C and contained 2.5 to 4 pmol of the indicated primer-template, either unlabeled or radiolabeled using [γ-^32^P]ATP (PerkinElmer) and T4 polynucleotide kinase (New England Biolabs) or fluorescently labeled during synthesis as indicated; WT or mutant Pol, with or without a 2-fold molar excess of UL44ΔC290 (58) (kindly provided by Gloria Komazin-Meredith), as indicated; and polymerase buffer (50 mM Tris [pH 8.0], 1 mM dithiothreitol, 100 mM KCl, and 40 μg/ml bovine serum albumin). Reactions were initiated by adding MgCl_2_ to 10 mM and, after incubation at 37°C for the times indicated, quenched using equal volumes of stopping buffer (0.05% bromophenol blue, 0.05% xylene cyanol, and 10 mM ethylenediaminetetraacetic acid [EDTA] in formamide) for polymerase and exonuclease reactions or 25 mM EDTA, 1% SDS, 5 mM dATP plus 5 mM dAMP for the idling assay, or 100 mM EDTA in 80% formamide for assays comparing extension of DNA or RNA primers on FAM-labeled primer-templates.

Apparent *K_m_* and *k*_cat_ values for incorporation of dGTP and GCV-TP by WT Pol or the mutant Pol A987G were measured as described previously (14) using radiolabeled primer-template and conditions that meet the requirements for Michaelis-Menten kinetic analysis. The reactions were determined to be linear for 15 min using GCV-TP as the substrate and were stopped at 12 min, while those using dGTP as the substrate were linear for 10 min and stopped at 5 min. Kinetic parameters were measured using previously described methods (14, 28). Apparent *K_i_* values were determined using a similar assay but with unlabeled primer template T1 and radiolabeled dGTP, whose incorporation was monitored using a filter-based method, as described previously (59).

Exonuclease assays were performed using polymerase buffer without any dNTPs added and 0.25 μM primer-template radiolabeled at its 5′ end, and, unless otherwise stated, 30 nM WT or mutant enzyme in the presence of a 2-fold molar excess of UL44ΔC290.

Full-length DNA extension by WT Pol and A987G Pol on primer-template T1 was assessed as described previously (14), with some modifications. Reactions were performed in polymerase assay buffer and contained 0.25 μM ^32^P-labeled primer-template T1, each enzyme at 7.2 nM or 8.4 nM, a 2-fold molar excess of UL44ΔC290, 25 μM GCV-TP, and 25 μM dCTP/dATP/dTTP.

To assess single-nucleotide extension on various primer-templates, similar polymerase assays were performed, with details about the enzyme concentrations, radiolabeled primer templates, and the incorporated single nucleotides or nucleotide analogs provided in Table 2.

To assay full-length extension using a DNA primer on FAM-labeled primer-template T5 by WT Pol or A987G Pol, a 20-μl polymerase reaction was performed in the polymerase buffer described above, containing 20 nM enzyme, 40 nM primer-template T5, and 1 mM dATP/dTTP/dCTP/dGTP. A similar assay was applied to test the extension of an RNA primer on FAM-labeled primer-template T6 by WT Pol or A987G Pol, but each enzyme was preincubated with T6 at 37°C for 1 min before initiation of the reactions. A 2-fold molar excess of UL44ΔC290 was added to the same assay to test its effects on DNA or RNA primer extension by each enzyme. Assays of extension of DNA or RNA primers on FAM-labeled T5 or T6, respectively, using WT HSV-1 Pol were performed using the same conditions except that 25 mM HEPES (pH 7.5) and 25 mM NaCl were used instead of 50 mM Tris and 100 mM KCl, and reactions were initiated with 8 mM MgCl_2_ (33).

For all of the above-described assays, except measurements of apparent *K_i_*s for GCV-TP, the products were separated on a 20% denaturing polyacrylamide gel, followed by quantification of radiolabeled products using a phosphorimager (Bio-Rad) or FAM-labeled products using an Amersham Typhoon 5 biomolecular imager (GE Healthcare) with detection at 520 nm following excitation at 495 nm.

Idling assays used 720 fmol each enzyme with UL44ΔC290, unlabeled primer-template T3, and 250 pmol dATP, including 10 μCi [α-^32^P]dATP in the polymerase buffer based on previously reported methods (14, 60). The reactions were analyzed by thin-layer chromatography (TLC) on polyethyleneimine/cellulose (Sigma-Aldrich) in 0.1 M phosphate buffer (pH 7.0), followed by phosphorimager analysis.

### Analysis of homologous polymerase structures.

To find high-resolution structures with which to model the interaction of HCMV Pol with primer-template, we first searched the solved structures of family B DNA polymerase for those bound to primer-template. Of these, we then selected structures containing either of two DNA polymerases that allowed us to see the relevant parts of the ligands and were the closest homologs to HCMV Pol (61). These are yeast DNA polymerase δ bound to primer-template and incoming nucleotide (PDB entry 31AY) and RB69 bound to primer-template either with incoming nucleotide (PDB entry 1IG9) or without nucleotide in editing mode (PDB entry 1CLQ). The close homology of those structures to herpesvirus Pols was further validated by 5-sequence Clustal Omega simultaneous alignment and by the ability to superimpose the thumb domain of the HSV-1 Pol structure (15) in rigid-body superposition in a way that makes the topological correspondence obvious and permits assignment of residues that correspond to those in HCMV Pol. Figures were generated with PyMOL. The amino acid sequence alignment in Fig. 7a was generated using Clustal Omega.
